# Prevalence of human papillomavirus infection and phylogenetic analysis of HPV-16 E6 variants among infected women from Northern Brazil

**DOI:** 10.1186/1750-9378-9-25

**Published:** 2014-08-05

**Authors:** Bruna Pedroso Tamegão-Lopes, Edivaldo Costa Sousa-Júnior, Fabio Passetti, Carlos Gil Ferreira, Wyller Alencar de Mello, Rodrigo Vellasco Duarte Silvestre

**Affiliations:** 1 Laboratório de Papilomavírus, Seção de virologia, Instituto Evandro Chagas, Rodovia BR 316 km 07, Ananindeua, Pará, Brazil; 2 Instituto Nacional de Câncer (INCA), Clinical Research Coordination, Rua André Cavalcanti, 37, 20231-050 Rio de Janeiro, RJ, Brazil

**Keywords:** Prevalence, HPV, HPV-16, Variants

## Abstract

**Background:**

The main cause of cervical cancer in the world is high risks human papillomavirus infection (mainly represented by HPV-16 and HPV-18), that are associated to the development of malign transformation of the epithelium. HPV prevalence exhibits a wide geographical variability and HPV-16 variants have been related to an increased risk of developing cervical intraepithelial lesion. The aim of this study was to describe DNA-HPV prevalence and HPV-16 variants among a women population from Northern Brazil.

**Methods:**

One hundred and forty three women, during routine cervical cancer screening, at *Juruti* Project, fulfilled an epidemiological inquiry and were screened through a molecular HPV test. HPV-16 variants were determined by sequencing the HPV-16 E6 open reading frame.

**Results:**

Forty two samples were considered HPV positive (29.4%). None of those had abnormal cytology results. HPV prevalence varied between different age groups (Z(U) = 14.62; p = <0.0001) and high-risk HPVs were more frequent among younger ages. The most prevalent type was HPV-16 (14%) and it variants were classified, predominantly, as European (87.5%).

**Conclusions:**

HPV prevalence in our population was higher than described by others and the most prevalent HPV types were high-risk HPVs. The European HPV-16 variant was the most prevalent among HPV-16 positive samples. Our study reinforces the fact that women with normal cytology and a positive molecular test for high-risk HPVs should be submitted to continuous follow up, in order to verify persistence of infection, promoting an early diagnosis of cervical cancer and/or its precursors.

## Background

Human papillomavirus (HPV) is a large group of epitheliotropic viruses of more than 160 different types. HPVs are very successful infectious agents – inducing chronic infections that have no systemic sequelae, rarely killing the host and shedding large amounts of infectious particles for transmission [1]. However, a dozen of the 40 HPV types that infect the human body are associated with probable or definite oncogenic risk [2].

High-risk HPVs (hrHPV), which includes HPV type 16 (HPV-16), are etiological agents of cervical cancer [3-5], being the persistent of hrHPV infection, known as the integrated HPV DNA into host genome, in the presence of other environmental and host factors, a necessary step for the development of high-grade cervical intraepithelial neoplasia and invasive cervical cancer [6-9]. Cervical cancer is the third most common cancer in women and more than 85% of the global burden occurred in developing countries, causing 275,000 deaths in 2008, with 31,400 of those deaths occurring in Latin America [10].

HPV molecular variants are defined taxonomically based on L1 coding region of the DNA sequence [11]. The comparative nucleotide sequence analysis, that includes ORF E6, have shown that HPV-16 variants, which differ in nucleotide sequence by no more than 2% in the coding regions and until a 5% in the non coding region, evolved into six major phylogenetic branches: European (E), Asian (As), Asian-American (AA), African-1 (Af1), African-2 (Af2) and North-American (NA1) [5,12,13]. Instead only a small portion of HPV-16 infections persist, several studies from Europe and the Americas, based principally on the sequencing of E6 and/or the LCR, had suggested that HPV-16 variants can influence viral persistence and the development of cervical cancer [14,15].

The aim of this study was to describe HPV infection prevalence and related epidemiological variables, as well as, the HPV-16 variants circulating in a population of women from Northern Brazil.

## Results

Socio-demographic data was obtained for a total of 143 women. Data analysis revealed that these women had 34 years median age (SD ± 13.74 y; 95% CI 32-36), were married (48%), had basic education as the main school level (58%), had housewife occupation (35%), were not exposed to alcohol (58%) or to tobacco (86%), had no history of STD’s (Sexual Transmitted Diseases) (84%), had 16 years old (SD ± 2.9 y; 95% CI 15.5-16.5) as the median age of the first sexual intercourse and had a median of 10 sexual partners (SD ± 32.3 partners; 95% CI 4.3-15.3). The higher median observed on the variable number of sexual partners was probably influenced by data related to women that referred to be sex workers (n = 7). When these data was removed from analysis, observed media of sexual partners was 5.5 partners (SD ± 8.6 partners; 95% CI 4-7). There was no observed statistical association between the described variables and the occurrence of HPV infection in our population (p > 0.05) (Table 1).

**Table 1:** Distribution of socio-demographic characteristics of the 143 women included in this study

**Variable**	**N = 143 n (%)**	**p value**
**Marital status?**		
Married	69 (48)	0.4644
Single or live with partner	66 (46)	
Widow	1 (1)	
Not informed	7 (5)	
**Scholarity?**		
Illiterate	4 (3)	0.8489
Literate	50 (35)	
Basic Education	83 (58)	
Not informed	6 (4)	
**Occupation?**		
Housewife	50 (35)	0.1789
Government employee	17 (12)	
Student	11 (8)	
Husbandman	11 (8)	
Sex worker	7 (5)	
Teacher	5 (3)	
Retired	4 (3)	
Nursing assistant	4 (3)	
Others	21 (14)	
Not informed	13 (9)	
**Alcohol?**		
Yes	54 (38)	0.3405
No	83 (58)	
Not informed	6 (4)	
**Exposition to tobacco?**		
Yes	13 (9)	1.000
No	123 (86)	
Not informed	7 (5)	
**History of STD?**		
Yes	11 (7.7)	1.000
**Which?**	Condyloma: 3 (27.5)	
	Syphilis: 3 (27,5)	
	Vaginal discharge: 1 (9)	
	Gonorrhea: 1 (9)	
	Trichomoniasis: 1 (9)	
	Not informed: 2 (18)	
No	120 (84)	
Not informed	12 (8.3)	
**Use of condom in sexual intercourse?**		
Yes	30 (21)	
No	107 (75)	0.6536
Not informed	6 (4)	

All of the 143 samples included in this study had normal cytology results. 23 HPV negative samples (23/101; 23%) had exhibited no amplification of internal control gene and were considered inadequate for subsequent analysis. Linear Array HPV Genotyping Test® (Roche Molecular Systems, Alameda, CA) results revealed that 78 samples were negative (78/120; 65%) and 42 were positive (42/120; 35%) for HPV infection. Among the HPV positive samples, the high risk HPV-16 was the most prevalent type (14%), followed by HPV-52 (9.5%) and HPV-45 (7%). Among low risk HPV types HPV-62 (7%) was the most frequent. The others high and low risk HPV types were identified in low frequencies (<2,5% each). From those HPV-16 positive samples, six were HPV-16 single infection (14.3%) and five were multiple infections that included HPV-16 (12%) (Table 2).

**Table 2:** Distribution of HPV types according to Array HPV Genotyping Test® results

**HPV types (N = 42)**	**n (%)**
**High risk**	
16	6 (14)
52	4 (9.5)
45	3 (7.1)
51	2 (4.7)
18	1 (2.4)
31	1 (2.4)
58	1 (2.4)
70	1 (2.4)
**Low risk**	
62	3 (7.1)
6	1 (2.4)
54	1 (2.4)
55	1 (2.4)
61	1 (2.4)
72	1 (2.4)
GTIS39	1 (2.4)
**Multiple**	
16 + 18	1 (2.4)
16 + 31	1 (2.4)
16 + 59	1 (2.4)
16 + 31 + 52	1 (2.4)
16 + 51 + 56	1 (2.4)
52 + 58	1 (2.4)
56 + 39	1 (2.4)
61 + 81	1 (2.4)
35 + 52 + 59	1 (2.4)
45 + 51 + 62	1 (2.4)
45 + 55 + 61	1 (2.4)
45 + 58 + 61	1 (2.4)
11 + 53 + 84 + GTCP6108	1 (2.4)
**Indeterminate**	1 (2.4)

**Figure 1:**
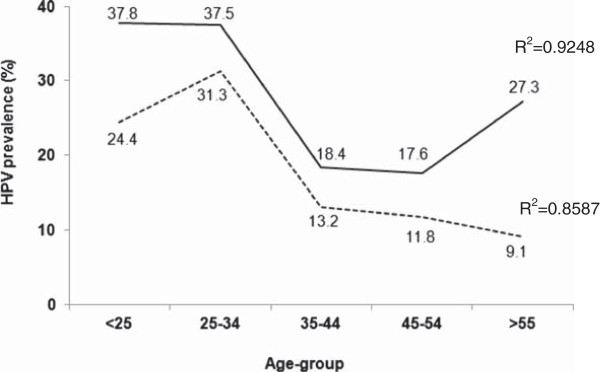
**Age-group and HPV infection prevalence.** High risk HPVs – dotted line; All HPV types – full line.

**Figure 2:**
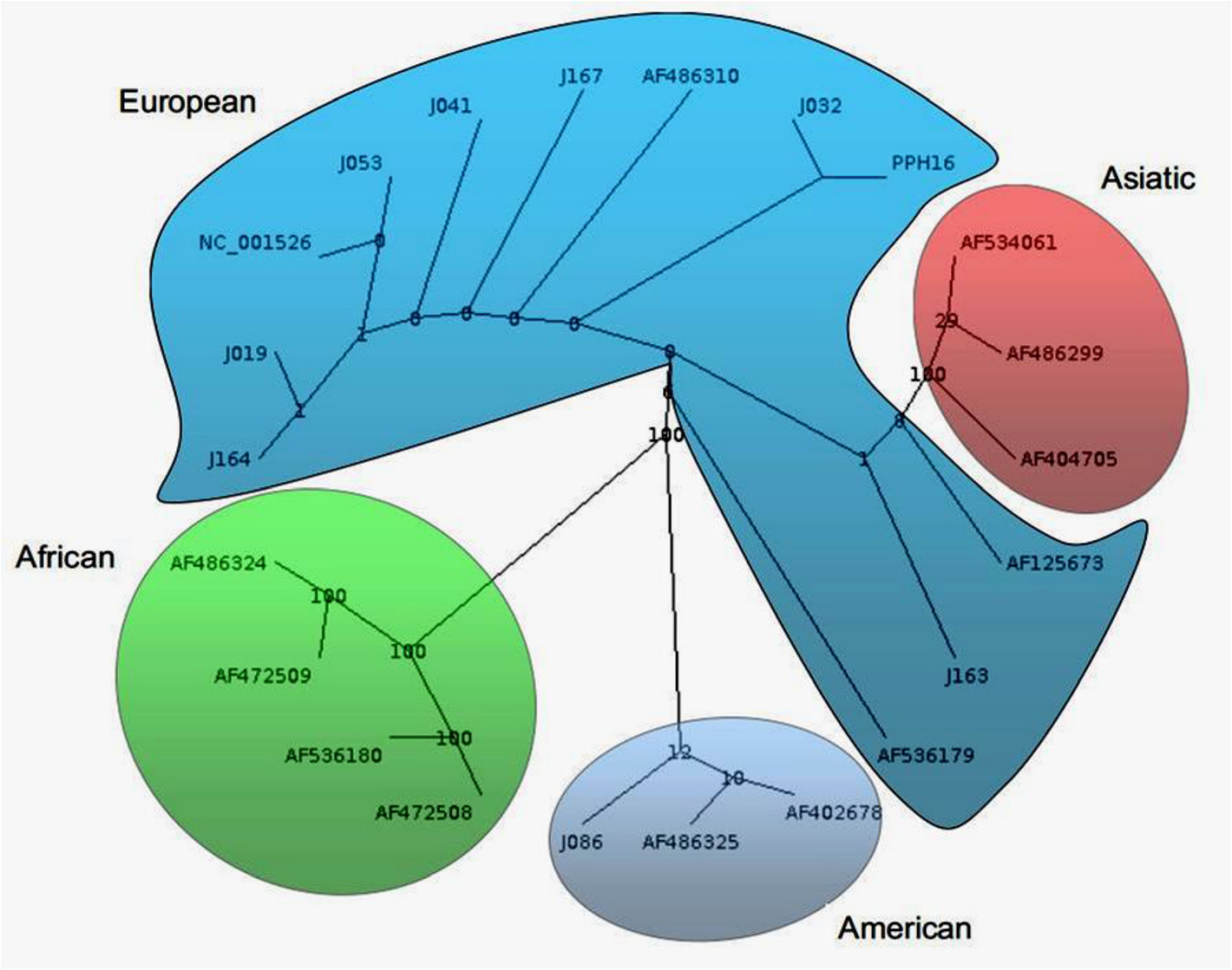
**HPV-16 phylogenetic tree based on E6 sequencing.** A phylogenetic tree was constructed using the RaxML program. This is a bootstrapped (1.000 replicates) consensus Maximum-Likelihood tree. PPH16 – GenBank accession number K02718 (HPV-16R). American branch (light blue) includes North American (NA) and Asian-American (AA) variants.

There was a variation of HPV prevalence between different age groups (Figure 1) and age was associated with HPV prevalence (Z(U) = 14.62; p = <0.0001). HPV of low and high risk was more prevalent among women at younger ages (<25 years) (37.8%; 95% CI 31.6%-44%) and hrHPV types were more prevalent among 25-34 age-group women (31.3%; 95% CI 26.9%-36.4%) (Table 3).Phylogenetic analysis was performed for a total of 8 samples among the HPV-16 positive samples (73%). The phylogenetic tree segregated into four major branches that could be recognized as European (EUR), Asian (As), African (including Afr1 and Afr2) and American (including Asian-American and North-American) lineages. The HPV-16 variants were classified as European (87.5%) and American (12.5%) (Figure 2).

**Table 3:** Distribution of age-group according to HPV infection

**Age at sample collection**	**Nº of women**	**HPV + (%) (CI 95)**	**hrHPV + (%) (CI 95)**	**p**
<25	45	17 (37.8) (31.6%-44%)	11 (24.4) (20.4%-28.4%)	<0.0001
25-34	32	12 (37.5) (31.4%-43.6%)	10 (31.3) (26.9%-36.4%)	
35-44	38	7 (18.4) (15.4%-21.4%)	5 (13.2) (11%-15.4%)	
45-54	17	3 (17.6) (14.7%-20.5%)	2 (11.8) (9.9%-13.7%)	
>54	11	3 (27.3) (22.8%-31.8%)	1 (9.1) (7.6%-10.6%)	
**Total**	143	42 (29.4)	29 (20.3)	

Legend: hrHPV – high risk HPV.

## Discussion

In the present study we found a 29.4% HPV prevalence between women without abnormal cytology screened by Pap Smear Test, at *Juruti*, Northern Brazil. This prevalence was higher than that described by SanJosé et al [16], in meta-analysis of a systematic literature review of HPV prevalence among women with normal cervical cytology worldwide, where global and South America prevalence’s were 10.4% and 12.3%, respectively. As well as, that described by Bruni et al [17], in meta-analysis of one million women with normal cytological findings, where global and South America prevalence’s were 11.7% and 15.3%, respectively. A broader range of HPV prevalence (from 10.4% to 24.5%) has been described in Brazilian studies including women with normal cytology. Our result was similar to that described by the *Latin America Screening Study* (24.5%) [8] and to data related to Amazonas State (29.2%) [18]. However, it was lower than described in others Brazilian studies including women populations from Rio Grande do Sul (34%), São Paulo (58.4%), Distrito Federal (62%) and Rio de Janeiro (63.6%) [19-21].

A marked variation of HPV prevalence among age groups was observed in our population. Women with less than 25 years and comprehend between 25-34 years were more predisposal to have a positive HPV-DNA test, including for hrHPV types. Some studies in Central and South America have demonstrated a first peak prevalence of HPV infection in women below age 25 and a second peak after age 55 [22,23] which corroborates our data. The first peak suggest that women acquire infection in the first few years after become sexually active and the second peak suggest that middle-age women harbor long-term HPV persistent infections or new incident infections, which might also reflect changes in hormonal or immunological status [24,25].

HPV-16 was the most prevalent type observed in our study (14.3%), followed by HPV-52 and HPV-45. The most prevalent HPV types described in women with normal cytology are HPV-16, -58, -31, -18, -45 and -52, however, it is well documented that this prevalence is lower than that observed among women with HSIL (high-grade lesions) and SCC (squamous-cell carcinoma) [26]. HPV-16 prevalence in our population was higher than that described in Natal (Northeast Brazil) (12.7%) [27] and lower than that observed in Rio Grande do Sul (19.6%), Rio de Janeiro (19%), São Paulo (18.2%) and Amazonas (31.8%) [18-21].

Phylogenetic analysis based on E6 sequences of eight HPV-16 isolates allowed the identification of seven European and only one non-European variants. A previous study realized in Belém (Northern Brazil) exhibited a higher prevalence of Asian-American (43.2%) and European (42%) variants among women with cervical cancer [28]. Previous data suggests that women harboring non-European HPV-16 variants have an 2- to 9- fold increased risk of cervical cancer [29,30], however, it has been reported that HPV-16 European variants persists longer in white women [31-33].

Genital HPV infection is one of the most common sexually transmitted infections worldwide. It has been estimated that approximately 10% of women worldwide with normal cytological findings carry a detectable cervical HPV infection, depending on the HPV testing technology, study size, and the age groups and geographical region studied [34]. It is necessary to emphasize that women with one positive DNA test for HPV-16, as well as, for others hrHPV, need to be followed-up to confirm persistent infection, to monitor and to avoid late diagnosis of possible cervical cancer and its precursors, since it is well documented that HPV-16 and HPV-18 account for 70% of all cervical cancer cases worldwide. This will be only achieved through consistent cervical screening associated with alternative screening tools as molecular techniques.

A major limitation of the present study is represented by the modest sample size and by the absence of a follow-up period to confirm persistence of HPV infection or clearance through timeline, as well as, the lack of ethnicity data, which was expected to be associated with HPV-16 variants. Another limitation was related with no amplification of internal control (β-globin gene) between 23 cervical samples on the Linear Array HPV Genotyping Test®. Those results were probably caused by inadequate sample cellularity (yield DNA specimens), as the DNA extraction technique was held as a constant, with DNA isolated using the same commercial kit.

## Conclusion

HPV prevalence (29.4%) in a population of women with normal cytology from *Juruti* (Northern Brazil) is higher than described by others with the most prevalent HPV types being hrHPVs (HPV-16, -52 and -45). The European HPV-16 variant (87.5%) is the most prevalent among HPV-16 positive samples. HPV-16 is defined as a human carcinogenic agent and is associated with cervical cancer among carriers. Despite being descriptive, the present study reinforces the necessity to follow up women with normal cytology, meanwhile, with a positive molecular test for hrHPVs, to determine persistence of infection and to promote early diagnosis of cervical cancer and its precursors.

## Methods

### Study population

Samples were collected from patients included on the *Juruti* Project, a research project designed to investigate the impact of migratory flow on the incidence and prevalence of sexual transmissible diseases. *Juruti* is a county located in the West Region of the State of Pará that had an estimated population of 34.338 individuals in the year of 2007. The main economical activities of *Juruti* are agriculture and mining and this county is marked by migratory flow of people from the States of Amazonas (Northern Brazil), Maranhão and Ceará (Northeastern Brazil).

During fifteen days of March (2007) women participating on the *Juruti* Project were offered systematic cervical cancer screening (Pap smear test) combining conventional cytology through Cytobrush Plus Cell Collector® (Coopersurgical, Trumbull, Connecticut, US) for optimal collection of exfoliated cells and a commercialized version of the line blot assay – Linear Array HPV Genotyping Test® (Roche Molecular Systems, Alameda, CA) – when they had their routine cervical smear performed in a public hospital. The *Juruti* Project has been approved by the *Instituto Evandro Chagas*, statement nº30/05, CAAE 0013.0.072.000-06DC-2008-374. All women were informed and gave their written consent to participate in the study.

### HPV cytological and molecular analysis

After samples collection cytobrush was immersed into 15 ml falcon tubes with 3ml of phosphate buffer saline (PBS: 137 mM NaCl, 10 mM Phosphate buffer, 2,7 mM KCl, pH 7.4) and kept at 4ºC according to the manufacturer’s instructions. At the HPV laboratory the falcon tubes were submitted to manual agitation, followed by cytobrush discard and centrifugation of the liquid per fifteen minutes (1200 rpm). Supernatant was discarded and cell pellet was resuspended in 3 mL of PBS solution.

The conventional cytology results were classified according to the recommendations of Brazilian Ministry of Health and Brazilian Society of Cytology which is based on Bethesda’s definition [35] and were performed at *Juruti* by the local routine professionals. Cervical specimen DNA extraction was performed according to the Linear Array HPV Genotyping Test® kit protocol (Roche Molecular Systems, Alameda, CA), which can detect until thirty seven distinct HPV types in a unique reaction, which includes high and low risk and multiple infections. Only the strips that had an internal positive control test (β-globin gene) were considered proper to genotyping analysis.

The PCR and sequencing of the HPV 16 E6 gene (nt 46-256) was performed as described by Chopjitt et al [5]. The amplification of E6 fragment was confirmed as described before^y^. The obtained nucleotide sequences of the E6 region were edited and assembled using UGENE software. Sequences were globally aligned and compared with HPV variants sequences available at GenBank database (GenBank accession number: K02718, NC_001526, AF125673, AF536179, AF486310, AF472508, AF536180, AF472509, AF486324, AF404705, AF534061, AF486299, AF402678, AF486325) using MAFFT version 7 software. Phylogenetic tree was constructed and edited using RaxML version 7.0.4 (1.000 bootstraps) and Dendroscope version 3 software’s, respectively.

### Statistical analysis

Data were analyzed with the software BioEstat 5.3 [36]. Fisher test was used to compare categorical variables between two groups (HPV-infected and non-infected women). Wilcoxon-Mann-Whitney test and G test were used to verify a possible association between age and HPV prevalence. After plotting HPV prevalence and hrHPV prevalence among HPV-infected women, in each age-group, a polynomial fit was assessed as the best model for logistic regression.

## Competing interests

The authors declare that they have no competing interests.

## Authors’ contributions

BPTL performed the genotyping of HPV variants, data and statistical analysis and lead drafting of the manuscript. ESCJ participated in the HPV variant analysis and performed phylogenetic analysis. WAM is member of the HPV at *Instituto Evandro Chagas* and is one of the researchers of the *Juruti* Project. FP participated in the study design and data interpretation. CGMS participated in the study design and data interpretation. RVDS was responsible for study design, for the initial HPV-DNA investigation, is a researcher and member of the HPV group at *Instituto Evandro Chagas*. All authors read and approved the final manuscript.
